# Carbonated rankinite binder: effect of curing parameters on microstructure, strength development and durability performance

**DOI:** 10.1038/s41598-020-71270-w

**Published:** 2020-09-02

**Authors:** Agne Smigelskyte, Raimundas Siauciunas, Harald Hilbig, Marco Decker, Liudvikas Urbonas, Gintautas Skripkiunas

**Affiliations:** 1 Department of Silicate Technology, Kaunas University of Technology, Radvilenu pl. 19, 50270 Kaunas, Lithuania; 2 Centre for Building Materials, Technical University of Munich, Baumbachstraße 7, 81245 Munich, Germany; 3 Department of Building Materials and Fire Safety, Vilnius Gediminas Technical University, Sauletekio al. 11, 10221 Vilnius, Lithuania

**Keywords:** Materials science, Inorganic chemistry, Materials chemistry

## Abstract

Due to the high CO_2_-footprint of ordinary Portland cement (OPC), the search for alternative binders is now in a full swing. Rankinite—which is a hydraulically inactive material and low in calcium, is a real alternative to OPC, as it absorbs the harmful greenhouse gas from the air through carbonation hardening. Nevertheless, the carbonation hardening has not yet been fully clarified and sufficiently investigated. In this study we show that rankinite achieves a final strength exceeding 100 MPa at 45 °C and 24 h, whereby the binder is only ~ 50% carbonated. The reaction is diffusion limited while a dense layer of carbonation products around the rankinite grains hinders a higher degree of carbonation. The carbonation reaction could be fully characterized by spatially resolved microanalysis such as LA-ICP-MS, NMR and XRD. Finally, durability tests show the excellent suitability of the rankinite binder for a wide range of applications, making it an attractive material not only from an environmental point of view.

## Introduction

Concrete is the second largest processed commodity after water consumed annually by the population of Earth^1^. Due to such vast demand for the building materials, ordinary Portland cement (OPC) industry is responsible for over 5% of global anthropogenic greenhouse gas emissions, with almost equal amount of CO_2_ emitted to the atmosphere after production of one tonne of cement^2–4^. Accordingly, the scientific community is struggling to find the solutions for greenhouse gas mitigation and reduction of the negative effect of the cement production. Even though, in the past decades many options to alleviate the adverse effect of OPC production to the environment were proposed^5^, however, recent studies have shown that strategies like clinker substitutions, alternative fuels and/or improved energy efficiency alone will not be sufficient enough to meet the target CO_2_ reductions^6^. Thus, finding alternative cementitious materials with lower CO_2_ footprint than OPC is one the major challenges for the building material industry and the scientific community. At the moment, one of the most promising approaches is the production of low-lime calcium silicate cement (CSC)^7–11^. This type of binding material not only requires lower amounts of limestone but also has lower production temperature, thereby resulting in much lower CO_2_ emissions^12^. Moreover, such binders are environmentally amicable not only due to lower CO_2_ emissions, but also for the ability to permanently store CO_2_ in the concrete structure in their carbonation hardening process^13^. Implementation of such efficient carbonation technologies can potentially lead to cementitious materials becoming one of the largest global CO_2_ sequestration sectors^14^.

Rankinite—Ca_3_Si_2_O_7_—is one of such low lime calcium silicates that can be used as an alternative binder that gained more interest recently^7,15,16^. Since the CaO/SiO_2_ ratio of rankinite is almost twice lower than of ordinary cement, thus it requires lower amounts of calcareous raw materials. The fuel and energy requirements for this type of binder are also reduced since the synthesis temperature of rankinite is 200 °C lower than that of OPC clinker and it does not require any additional energy intensive processing, like rapid cooling^17^. Moreover, rankinite can be produced from the same raw materials as OPC clinker while the existing production plants are also suitable for its production, thus no major adjustments would be necessary. Most importantly, rankinite is a non-hydraulic binder that hardens in the CO_2_ atmosphere, resulting in the highly durable structure of calcium carbonates and silica gel^18^. Since carbonation is a diffusion limited process, it relies on many process environment and sample parameters that needs be taken into account and reconciled.

However, in the viewpoint of implementing such alternative binders in the global market, the priority in the cement research is to investigate material properties, e.g. strength, durability and etc. For an alternative binder to be competitive to OPC it needs to perform similarly or surpass the performance of the existing technologies. Thus, comparison of OPC and rankinite binder efficiency is of high importance.

Due to this, the aim of this work is to investigate rankinite binder carbonation hardening process and parameter effect on the compressive strength development and obtained concrete durability, resulting reaction products and microstructure using a multi-technic approach.

## Materials and methods

### Materials and sample preparation

Rankinite binder used in this work was synthesized from opoka (a sedimentary lime-silica rock) and limestone at 1,250 °C for 45 min; the details regarding the components and methods used for the binder synthesis can be found in a previous paper^17^. Other materials used are: CEN Standard sand EN 196-1 (Germany); Portland cement CEM I 42.5 R (Lithuania); 3% NaCl solution.

The paste samples were prepared by mixing the rankinite binder with distilled water to obtain water to solid (w/s) ratio of 0.125. The obtained moist powder was then moulded and compacted to form cylindrical samples of 36 × 36 mm^3^ dimensions. The compaction pressure used was 12.5 MPa. After formation the samples were immediately transported to a pressure reactor without any preconditioning and cured using pressurized CO_2_ (15 bar, 99.9% purity) at 25 to 55 °C for 2 to 48 h.

The mortar samples were prepared by mixing the rankinite binder with sand with two different binder to sand ratios of 1:1 and 1:3; water to solid (w/s) ratio was constant at 0.0625. The sample dimensions were the same as for the pastes and the samples were moulded and cured at the same conditions as the pastes also, but only for 24 h at 45 °C.

For durability determination, identical mortar samples of both rankinite binder and cement (OPC) were prepared (three of each). The mortars were prepared as follows: tiles with dimensions of 100 × 100 × 20 mm, binder to sand ratio 1:3, w/c = 0.35 (for OPC) and w/c = 0.25 (for C_3_S_2_) were pressed (compaction of 12.5 MPa) and cured at 15 bar for 24 h at 45 °C. Such sample dimensions were chosen in order to achieve a surface area between 7,500 and 25,000 mm^2^ (according to the standard EN 1338:2003+AC:2006). For durability determination, the samples were prepared by insulating all but one side, which was then poured over with 3% NaCl solution and exposed to freeze–thaw cycles, collecting the scaled material and calculating the average cumulative scaled mass.

*Carbonation curing* was carried out in a Parr Instruments (USA) pressure reactor, model 4,555 with a maximum working pressure of 131 bar, a volume—18.75 l, and a temperature range— − 10–350 °C. The reactor was first twice purged with CO_2_ gas up to ~ 2 bar and immediately depressurized to atmospheric pressure to eliminate the presence of air and afterwards the pressure of gaseous CO_2_ was increased (and decreased afterwards) by 2.5 bar/min to the required value.

After the curing, the sample obtained compressive strength was immediately determined. Four samples were used for the determination of the compressive strength. Few samples of each batch were left for further analysis (chemical characterization by digestion/ICP-OES, MIP, XRD, LA-ICP-MS, NMR, SEM/EDX) after drying at 100 ± 5 °C for 24 h.

### Test methods

The *formation of the samples* and determination of the *compressive strength* was performed according to EN 196-1 and EN 12390-6 respectively, using a universal testing machine FORM + TEST MEGA 10-400-50 (Germany) at a loading rage of 1 kN/s (for forming) and 1.5 kN/s (for compressive strength).

Spatially resolved *X-ray diffraction* (XRD) analysis was performed by a Bruker D8 Advance diffractometer using CuKα radiation (λ = 1.54 Å, 40 kV, 40 mA) in θ–θ configuration with a LynxEye XE-T silicon strip detector and an automatic divergence slit (fixed radiation spot of 1 mm). The measurement range was 5°–50° 2θ with steps of 0.03° 2θ with a measurement duration of 2 s/step. A cross section (d = 3 mm) of the original sample was prepared using a Buehler IsoMet 5,000 precision saw. A motorized sample stage (Bruker Compact UMC, software controlled XYZ positioning) was used for creating X-ray diffraction profiles along the cross section of the sample.

The porosity and pore size distribution of the materials was determined using *mercury intrusion porosimetry* (MIP) (AutoPore III, Micromeritics, USA). The samples were dried at 40 °C for 24 h before the measurements.

*Laser ablation inductively coupled plasma mass spectrometry* (LA-ICP-MS) was performed by ESI NWR 213 (ESI New Wave Research, USA) laser, providing 10 lines, each 60 µm broad and 20,000 µm long, lines and NexION 300D Perkin Elmer (USA) spectrometer with NexION ICP MS Software Version 1.5; carrier gas 0.7 l min^−1^ He + 0.92 l min^−1^ Ar. The following isotopes were measured: ^43^Ca, ^29^Si and ^13^C.

The ^*29*^*Si Nuclear magnetic resonance* (NMR) experiments were performed with a Bruker Advance 300 spectrometer (magnetic field strength 7.0455 T, resonance frequency for ^29^Si: 59.63 MHz) in MAS (magic angle spinning) mode using the single pulse technique (90° pulse). The samples were packed in 7 mm zirconia rotors and spun with 5 kHz. About 2000 scans were recorded for each spectrum with a repetition time of 45 s. The chemical shifts were set relative to external tetramethylsilane. The signal patterns of the spectra were deconvoluted with Bruker WINNMR software^19^.

#### Chemical determination of the elements

From the cut sample half cylinders, smaller pieces of material were removed with hammer and chisel and ground. After careful mixing, the sample material was subjected to a representative subsample of approximately 0.5 g. Together with 1.5 g of lithium metaborate the material was placed in a weighed platinum crucible and annealed at 1,000 °C for 0.5 h. The loss on ignition (LOI) of the crucible was determined by weighing the crucible including the melting tablet after cooling. The fused tablet was then ground, the resulting powder dissolved in 1 molar nitric acid and filled up to 100 ml with ultrapure water. The element concentrations were determined using inductively coupled plasma optical emission spectrometry (ICP-OES, Jobin Yvon Ultima II).

For the microstructure observations, the carbonated samples were cut in half using a precision saw. The cutting planes afterwards were sanded and polished using Tegramin-25 (Struers ApS, Denmark) equipment. For sanding, a SiC paper (#320) was used and for polishing, 9, 3, and 1 µm diamond suspensions and corresponding polishing substrates (Struers ApS) were used. To ensure surface electrical conductivity, the samples were coated with 20–30 nm carbon layer using a Q150T ES (Quorum Technologies Ltd.) system. Sample surface structure and elemental composition was investigated by *scanning electron microscopy* (SEM) using Helios Nanolab 650 (FEI) scanning electron microscope coupled with *energy dispersive X-ray* (EDX) spectrometer (Oxford Instruments, Xmax 20 mm^2^ detector, INCA 4.15 software). Surface images were obtained by recording secondary electrons at 5 kV accelerating voltage and 0.8 nA current. The elemental composition and distribution maps were obtained at 20 kV accelerated voltage and 3.2 nA current.

## Results and discussion

### Carbonated rankinite binder paste compressive strength development

**Figure 1:**
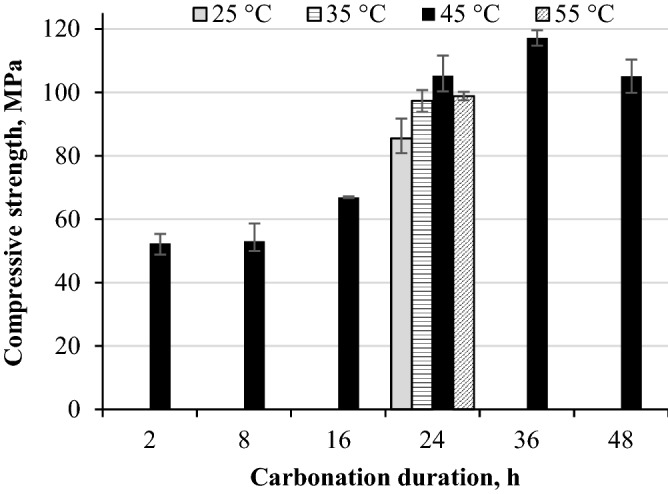
Compressive strength development of rankinite binder paste samples carbonated at 15 bar CO_2_ pressure at different temperature and duration.

Compressive strength development of the rankinite binder samples carbonated at different conditions is provided in Fig. 1. As can be seen in Fig. 1, samples carbonated for 2–8 h developed similar compressive strength of ~ 50 MPa. Even though the obtained sample strength is considerably high, however, the samples were visually not fully carbonated, since the fragments of the crushed samples showed a crumbly uncarbonated binder core. Carbonation duration extension up to 16 h led to further compressive strength development and increment of 26%, however, the samples were still not fully carbonated. Carbonation process extension to 24–48 h had a significant influence on the sample compressive strength development—the sample acquired strength exceeded 100 MPa and the samples were evidently fully carbonated, which was later on confirmed by further analytical techniques, the structure of the sample fragments was uniform. Since samples carbonated for 24–48 h seem to have reached similar strength values, it can be suggested that 24 h is a threshold point after which carbonation reaction is severely hindered, the samples are carbonated through the entire volume and the highest possible strength is achieved.

Since carbonation is a diffusion limited process^20^, temperature has a significant impact on the process development^21^. For this reason, rankinite binder samples were cured for 24 h at a temperature range of 25 to 55 °C. As shown in Fig. 1, the compressive strength developed with increasing temperature and reached the highest value at 45 °C, while further increase of the temperature up to 55 °C led to a 6% decrease in the sample strength value. This decrease of the compressive strength could be associated with the aragonite transformation to calcite at higher temperature that leads to an increase in free volume giving a rise to a microcrack formation that could adversely affect the compressive strength^22^, since presence of aragonite was later on detected by XRD analysis. Hence, the obtained results suggest that 45 °C is the optimum carbonation temperature for the rankinite binder samples to achieve the highest outcome.

**Figure 2:**
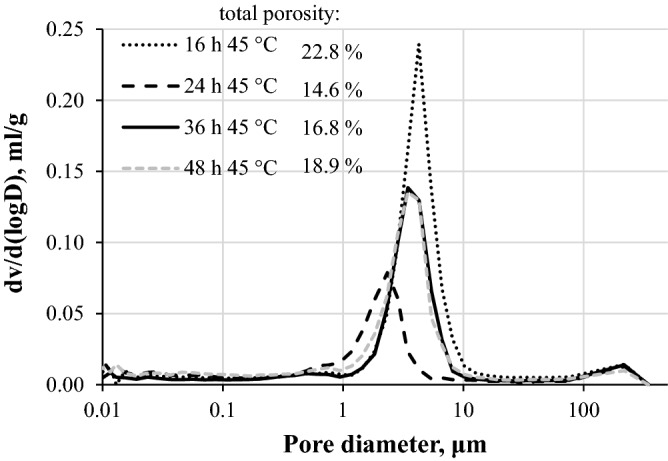
Total porosity and pore size distribution of carbonated rankinite binder samples.

The compressive strength results were supplemented with MIP analysis to determine the total porosity and the pore size distribution of the samples carbonated for different duration. It should be noted that the sample parts for the determination were taken from the outer (carbonated) part of the sample, ~ 10 mm from the edge as close to the same spot of each sample as possible to achieve comparable results. As can be seen in Fig. 2, the highest total porosity was determined in the samples carbonated for 16 h, while the pore size in these samples was also considerably higher (up to ~ 10 μm) than in those carbonated at prolonged durations (up to ~ 6 μm). 16 h carbonation results imply that the carbonation reaction was not complete, and the sample pores were not fully saturated with reaction products leading to higher porosity and thus lower compressive strength. However, the results of the samples carbonated at prolonged conditions are rather peculiar, since even though the samples reached similar compressive strength results, the obtained porosity ranged from ~ 15 to ~ 19%. These differences may be due to the different structure of the main reaction products, i.e. calcium carbonate and silica gel. Since calcium carbonate has three different polymorphs—calcite, vaterite and aragonite—the structure of them is rather different^23,24^, and this could impact the mechanical properties of the system. Silica gel, on the other hand, impacts the structure of the sample by different degree of polymerization that also could influence the mechanical properties. For this reason, further investigations were based on rankinite binder paste sample mineral composition development with the ongoing carbonation reaction.

### Carbonated rankinite binder paste mineral composition and microstructure development

#### XRD analysis

The XRD analysis was carried out to determine the mineral composition changes throughout the carbonated sample volume. The analysis was performed on the cross section of the sample, moving from the surface to the centre, where 9 points were analysed between 0.5 and 16.5 mm positions.

**Figure 3:**
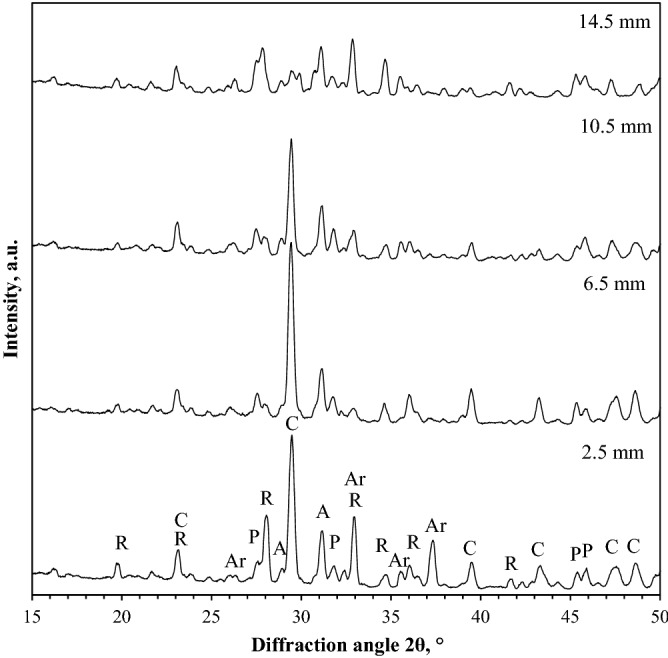
XRD patterns of rankinite binder sample carbonated for 8 h at different cross section positions. Indexes: R—rankinite, P—pseudowollastonite, A—akermanite, C—calcite, Ar—aragonite.

The cross section of the samples carbonated for the shorter duration (up to 16 h) appeared to be consisted of a brighter core of around ~ 10–15 mm diameter, surrounded by a transition zone of ~ 2 mm width and a darker outer layer. Selected results from the sample carbonated for 8 h are provided in Fig. 3.

Carbonation products, i.e. calcium carbonates, were detected within the outer layer, without even distribution. Carbonate peak intensities decreased, when moving to the centre of the sample. Rankinite was found in all of the measurement position and had higher concentrations in the inner core that evidently did not participate in the carbonation reaction. Pseudowollastonite was distributed similarly to rankinite, indicating that its carbonation undergoes the same path as rankinite, while akermanite was found evenly distributed throughout the entire cross section of the sample, showing that this mineral is non-reactive during the carbonation process; this was previously suggested by Ashraf et al.^7^, as well. Considering the newly formed calcium carbonates, two of the polymorphs—calcite and aragonite—were detected. Calcite showed a significant contrast in the concentration having higher concentrations in the outer layer, with the highest concentrations at 6.5 and 8.5 mm positions, while within the inner core its presence appeared to be minimal. Aragonite was only detected in the outer layer, with a maximum concentration at the 4.5 mm position. Presence of aragonite in the hardened part of the sample is most likely a result of a complex path of carbonation reaction, since the binder contains relatively high amount of magnesium which is known to impede calcite growth while favouring aragonite formation^25,26^.

Similar observations were made in the samples carbonated for 2 and 16 h, while mineral composition of the fully carbonated samples (24–48 h) appeared evenly distributed throughout the entire sample cross section, without any major discrepancies.

**Figure 4:**
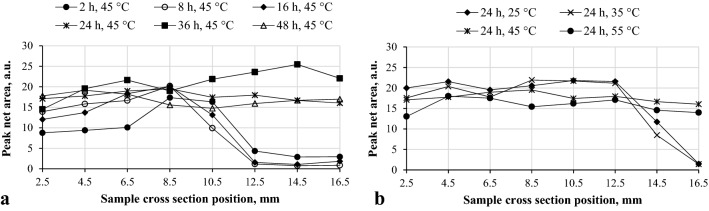
Calcite peak (*d-spacing* = 0.3027 nm, *2θ* = 29.48°) net area in positions throughout the sample cross section, dependence on duration (**a**) and temperature (**b**).

Since calcium carbonate in the form of calcite is the main mineral after the carbonation, its distribution throughout the sample cross sections is provided in Fig. 4, were only the main calcite peak (*d-spacing* = 0.3027 nm, 2*θ* = 29.48°) net area was taken into account.

As can be seen in Fig. 4, calcite formation and distribution in the sample cross sections highly depends on the process parameters. In the samples that were not fully carbonated, calcite seems to reach a maximum at a 8.5 mm position, considering the duration, or 12.5 mm, considering the temperature, after which it drops down to a negligible concentration. Considering the duration, the obtained results, further confirmed that only samples carbonated for 24 h and more were fully carbonated, since calcite distribution is rather even, throughout the cross section of the sample (Fig. 4a). While considering the temperature influence, only samples carbonated at > 45 °C were fully reacted (Fig. 4b). Evidently, samples carbonated for 36 h reached a higher carbonation degree, since the calcite peak net area, and thus intensity, is much higher than in other samples. This is most likely an explanation for the highest compressive strength (Fig. 1), since calcite is believed to be the main component responsible for the development of mechanical strength.

Even though, calcite distribution in different samples is rather contrasting, however, all of the samples seem to have reached a similar carbonation degree in the 6.5–8.5 mm positions. This may be the point in the sample volume, at which the carbonation process switches from phase-boundary, i.e. formation of the reaction products, controlled to a diffusion controlled process. It was already previously suggested that in the beginning, the carbonation reaction occurs very rapidly and is mostly dependent on the nucleation of the reaction products^27^. The second stage, however, is believed to be diffusion controlled, since at some point of the reaction, the layer of the reaction products becomes so thick that the CO_2_ diffusion through this layer becomes the limiting step for further reaction development^28^. Also, prior to carbonation, the pores of the sample are filled with water and air, with a pressure of 1 bar. During the carbonation, CO_2_ gas comprises the air to a pressure of 15 bar to the middle of the sample. Thus, the middle of the sample has low CO_2_ concentration, surrounded by a region of pure CO_2_. The obtained results suggest that 6.5–8.5 mm position may be a border of this condition. The carbonation of the inner part may now happen via slow diffusion that is temperature dependent.

#### LA-ICP-MS analysis

**Figure 5:**
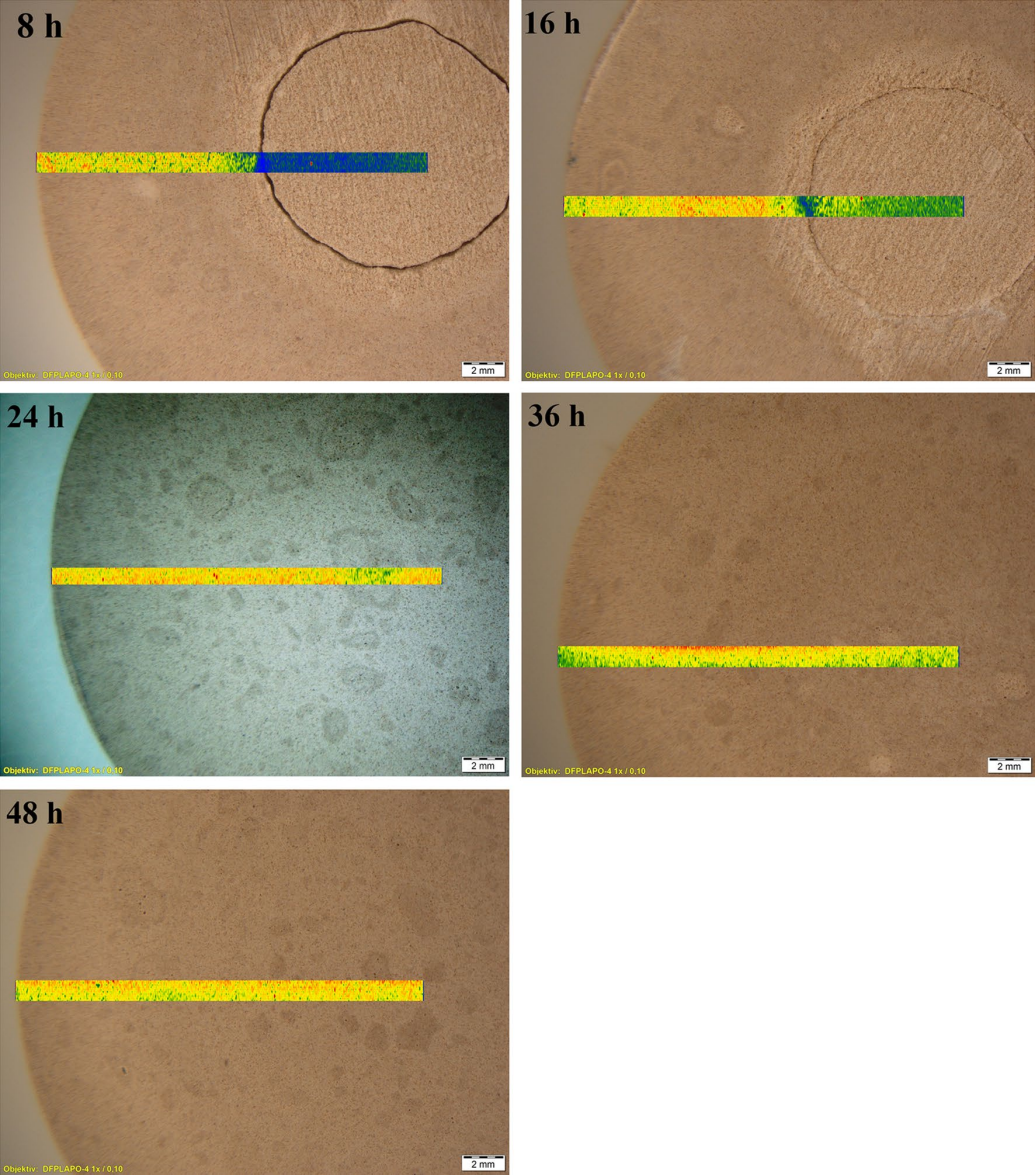
Carbonated (at 15 bar, 45 °C) rankinite binder sample cross sections with plotted carbon distribution (colour code from low to high: purple–blue–green–yellow–red).

Carbonate distribution in the sample cross sections was also investigated by LA-ICP-MS. The obtained results are provided in Fig. 5, where sample cross sections with plotted carbon distribution are shown. The element distribution is colour plot based, where purple represents the lowest intensities, while red represents the highest signal intensities.

As can be seen in Fig. 5, the obtained results further support previous analysis results that only the samples carbonated for 24 h and longer were fully carbonated and the reaction products are evenly distributed in the entire sample cross section.

Chemical composition analysis was carried out to further analyse the carbonated samples. The obtained results, where unreacted rankinite binder composition is also provided as a reference are shown in Table 1.

**Table 1:** Chemical composition of uncarbonated and carbonated rankinite binder samples.

Composition (wt%)	Uncarbonated rankinite binder	8 h carbonation outer part	8 h carbonation inner part	48 h carbonation
Total carbon	–	3.60	0.41	4.23
LOI 1,000 °C	–	15.34	2.64	17.51
Na_2_O	0.21	0.08	0.09	0.07
K_2_O	0.62	0.49	0.43	0.46
CaO	49.48	42.17	49.03	41.19
MgO	2.79	1.67	1.93	1.55
Fe_2_O_3_	1.28	1.45	1.70	1.43
Al_2_O_3_	2.34	1.69	1.92	1.59
SiO_2_	42.49	38.27	44.49	37.61
P_2_O_5_	0.09	0.09	0.10	0.09
SO_3_^2−^	0.20	0.22	0.13	0.25
TiO_2_	0.09	0.09	0.10	0.09
BaO	0.01	0.01	0.01	0.01
SrO	0.09	0.09	0.09	0.08
MnO	0.02	0.02	0.02	0.02
Cr_2_O_3_	0.01	0.01	0.02	0.01

Two of the carbonated samples were taken into account—8 h as an example of a non-fully carbonated sample and 48 h as a reference of a fully carbonated sample. As can be seen in the provided Table 1, composition of the sample carbonated for 8 h highly contrast comparing the inner and outer parts. The composition of the inner part of the sample matches the composition of the uncreated rankinite binder, further indicating that the core of the sample was in fact not reacted. However, carbonated samples contained loss of ignition (LOI), that was due to decomposition of carbonates and moisture, most likely tarped in the sample pores or silica gel structure, differing from raw rankinite binder. Recalculating the measured total carbon amount to carbonates and deducting it from the LOI results, the mass loss due to moisture was equal to 2.14 and 1.14%, in the outer and inner part of the 8 h sample, respectively. Furthermore, the total amount of carbon in the outer layer is around nine times higher indicating a much higher carbonation degree. Also, the composition of the 8 h sample outer part is very similar to a 48 h sample composition. Since, as previously determined, sample carbonated for 48 h chemical composition was rather evenly distributed, the total determined carbon amount reached 4.23%, which is equal to 35.25% of calcium carbonate, based on the molar mass. Maximum amount of calcium carbonate that can form during the carbonation of this specific rankinite binder was calculated in a previous study^29^ and was equal to ~ 85%. However, it is evident, that less than half of this amount has actually formed after carbonation for the longest duration. This suggest that as the coating of reaction products around the surface of the unreacted rankinite particle becomes thicker, the particle is surrounded by a densified layer, isolating the unreacted particle from further reaction and thus limiting the carbonation extent. Due to this, a considerably large part of the binder particles remains unreacted, preserved by a dense layer of carbonation reaction products that CO_2_ is no longer able to penetrate. This leads to the conclusion that even though the sample seems to be fully carbonated throughout the entire volume, only half on the binder mass has actually reacted. Nonetheless, the microstructure of the formed reaction products appears to sufficiently contribute to enhanced compressive strength. Summarizing the obtained results, it can be seen that carbonate formation proceeds insignificantly after 24 h at temperatures higher than 45 °C, since neither the chemical composition nor the compressive strength is significantly altering. At this time, the reaction products have most likely reached a point at which no further reaction can proceed, and the carbonation process is impeded. These results are in a good agreement with the literature reports that have also stated that irrespective of the type of binder, once a certain degree of carbonation has been reached, the further diffusion of CO_2_ is severely hindered by previously formed reaction products and reduced porosity and the maximum carbonation degree that can be reached was found to be around 40%^27^.

#### ^29^Si MAS NMR analysis

**Figure 6:**
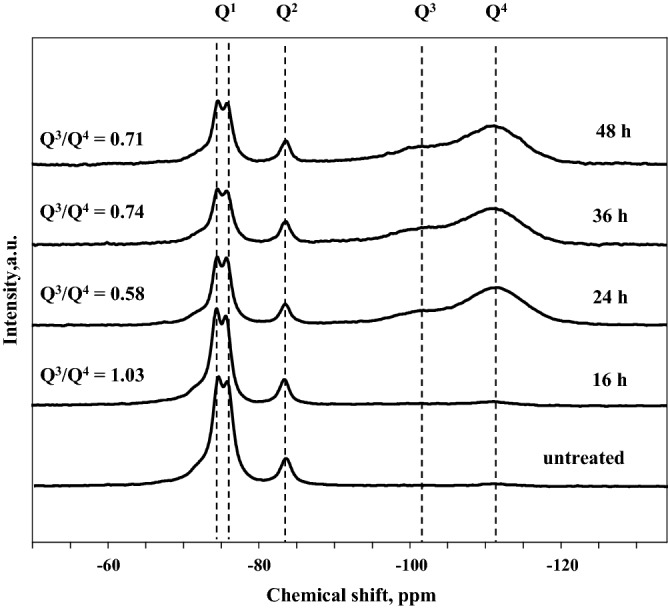
^29^Si MAS NMR spectrum of untreated and carbonated for 16, 24, 36, and 48 h at 15 bar and 45 °C rankinite binder samples.

However, the structure of the carbonated sample depends not only on the formation of the calcium carbonates but on the formation and polymerization of the silica gel as well. Silica gel formation and development was investigated by ^29^Si MAS NMR and the obtained results are provided in Fig. 6. The NMR spectrum of unreacted rankinite binder (Fig. 6, *untreated*) contained three major sharp peaks, two of which seem to be overlapped: at − 74.5 and − 75.8 ppm, and third the peak at − 83.6 ppm. According to the literature^7,18,30^ the first two peaks are assigned to Q^1^ species and are assigned to rankinite, since this mineral consists of an array of Si_2_O_7_^6−^ groups linked by Ca atoms^31^ while the third peak is assigned to Q^2^ species and is assigned to pseudowollastonite due to its chain silicate structure^30^. The intensities of the peaks at − 74.5 and − 75.8 ppm decreased with prolonged carbonation. Along with the carbonation reaction, new peaks at around − 101 and − 111 ppm appeared. The peak at − 101 ppm is attributed to the hydroxylated surface sites of the silica gel and is assigned to Q^3^ species, while the peak at − 111 ppm is attributed to the Q^4^ sites of a polymerized 3D network of the silicate tetrahedrons^18,32,33^. Ashraf et al.^7^ suggests that the presence of cross linked Q^3^ species indicates that the silica gel formed after the carbonation reaction is a Ca-modified silica gel. After 16 h carbonation the peaks at − 101 and − 111 ppm are of similarly low intensity as those of uncarbonated rankinite, whereas with prolonged carbonation (24, 36, and 48 h) the intensity of these peaks is much higher indicating that carbonation caused a significant formation of Q^3^ and Q^4^ silicate species. Although, all the carbonated samples still contained considerably high fractions of Q^1^ and Q^2^ species, these might not only be from the uncarbonated binder but from formation of C–S–H like phase^34^. However, since it was previously determined that only a half of the binder particles reacts with CO_2_, the remaining peaks are most likely due to the unreacted binder particles.

The degree of polymerization of the silica gel can be described by calculating the ratio of integrated areas of Q^3^/Q^4^, where lower values of Q^3^/Q^4^ represent higher fraction of fully condensed silicate clusters and therefore, higher degree of polymerization^18^. As the silicate condensation reaction proceeds, the amount of partially condensed silica tetrahedra (Q^3^ species, with three bridging and one non-bridging oxygen negatively charged) decreases and that of fully condensed (Q^4^ species, with four bridging oxygens) increases^35^. The silica tetrahedra are fully condensed by their four corners in a lattice extending over the three directions of space.

For samples carbonated for 16 h this ratio was equal to 1.03, while prolonged carbonation duration led to a decrease in the ratio value to 0.58, 0.74, and 0.71 for 24, 36, and 48 h respectively. It is evident that the highest degree of silica gel was obtained by the samples carbonated for 24 h. However, these does seem to correlate with compressive strength development, since the highest strength was gained by the samples carbonated for 36 h, while the samples carbonated for 24 and 48 h reach similar strength values. Even though, the compressive strength and silica gel polymerization values of all samples carbonated for the longer durations—24, 36, and 48 h—are rather similar, and fall into deviation values.

#### SEM analysis

**Figure 7:**
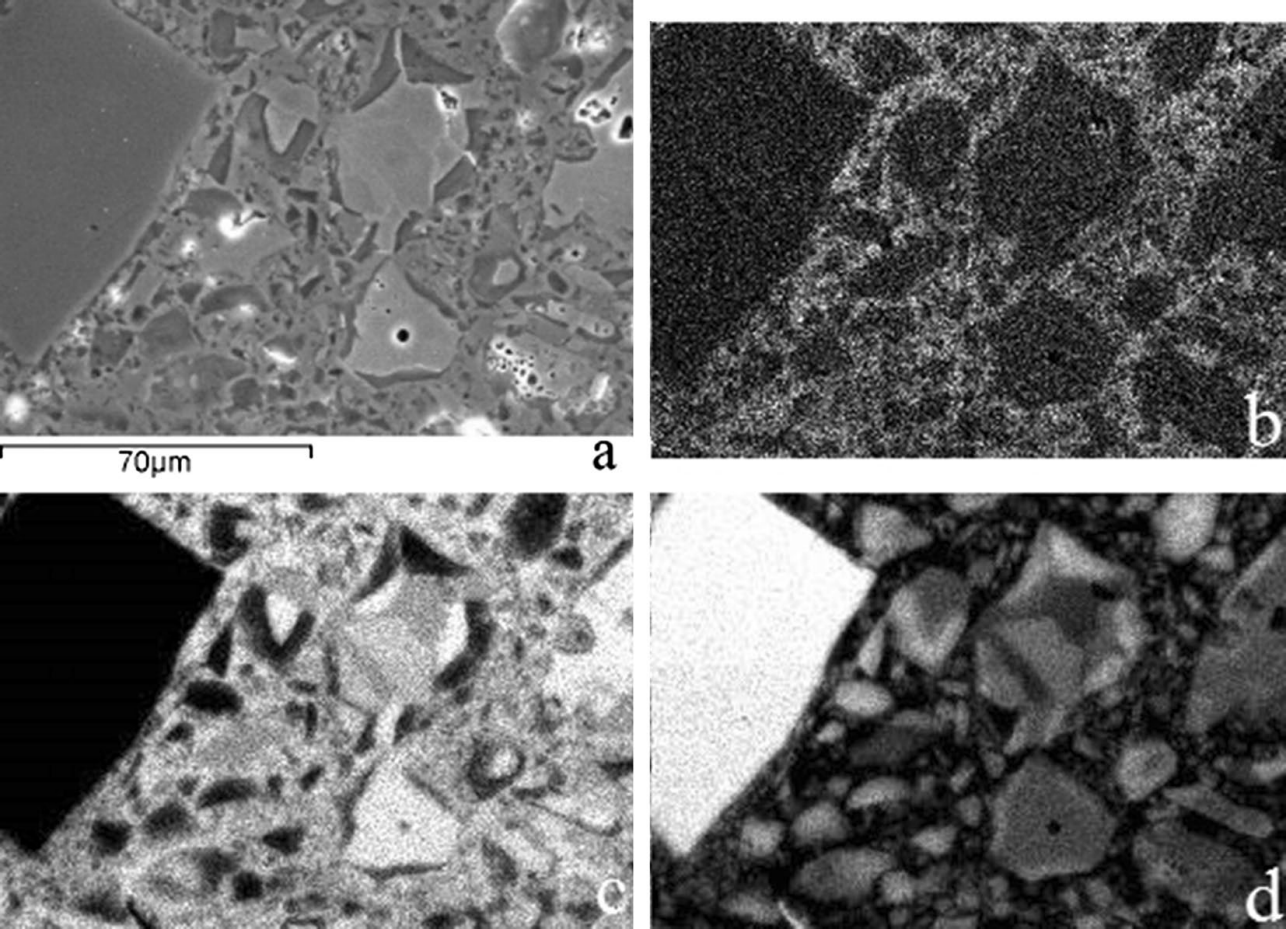
BSE image (**a**) of the carbonated rankinite binder sample (for 24 h at 45 °C) in the fully carbonated area near the edge with mapping of the element distribution: (**b**) C, (**c**) Ca, (**d**) Si (dark: low content, bright: high content).

The microstructure of the carbonated binder samples was investigated by SEM analysis, collecting backscattered electron (BSE) images and mapping the elemental distribution. Figure 7a show a BSE image of the sample carbonated for 24 h at 45 °C with the mapping of the prevailing elemental (C, Ca, and Si) distribution provided in Fig. 7b–d. Four different sites were identified based on the scale of the grey colour and the obtained element mapping results: the darkest grey regions are attributed to the silica gel, while the brightest grey is attributed to the unreacted binder particles, calcium carbonate was described by the medium light grey in between the large particles, and the pores, although easily visible, due to better phase contrast are in bright white.

Silica gel seems to be distributed as a rim around the unreacted (or partially reacted) binder particles with a dimensions of around 1–5 μm. This formation of silica gel around the binder particles was also previously reported by Ashraf et al.^7^. Calcium carbonates, on the other hand, seem to fill the spaces between the particles without any specific dimensions and are distributed rather evenly throughout the volume. The mapping also showed, that in the areas of silica gel, the amount of Ca is rather low, indicating that the silica gel may contain only insignificant amount of calcium in its structure.

From the obtained images (Fig. 7), the microstructure of the carbonated material was determined to be considerably porous, with pore diameters from 50 μm to less than 5 μm. It can concluded that the first layer surrounding the unreacted binder particle is silica gel, followed by the layer of calcium carbonate filling the pores and thus densifying the microstructure of the material. However, as previously mentioned, at some point, the layer of carbonation reaction products around the unreacted binder particles becomes so thick, it obstructs CO_2_ from further diffusion to the unreacted particles thus hindering further carbonation and restricting the material from additional strength development.

### Carbonated rankinite binder mortar compressive strength development and durability performance

Since in practice, concrete products are usually produced from binder and sand mixtures, this part of the research was dedicated to investigate the rankinite binder mortar compressive strength and durability development.

**Figure 8:**
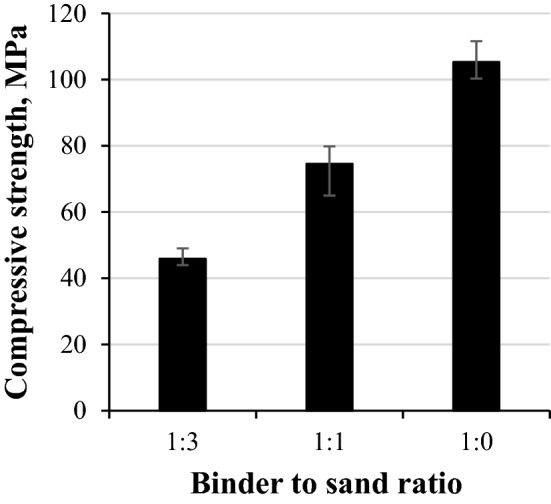
Compressive strength development of rankinite binder samples carbonated for 24 h at 15 bar and 45 °C with different binder to sand ratio.

Thus, mortar samples with two different binder to sand ratios—1:1 and 1:3—were prepared. These samples were cured at 15 bar for 24 h at 45 °C and sample compressive strength obtained is provided in Fig. 8. As can be seen, mortars reached significantly lower strength results compared to the paste samples. The mortar sample with a highest sand content (binder/sand 1:3) reached a compressive strength of ~ 45 MPa visible in Fig. 8. In comparison to the mortar sample with binder/sand ratio of 1:1 compressive strength was 40% lower, while in comparison to the paste sample (binder/sand 1:0) strength value was less than half as high. This considerable compressive strength improvement with lower sand ratio is associated with the sample microstructure. It is evident that without the aggregates, the structure of the sample is much denser and even in spite of this, samples were able to reach full carbonation. Due to the absence of sand, the sample is solely composed of the binder particles that later on recrystallized to calcium carbonate and silica gel, thus making the structure much more enhanced. Mortars with lower ratio of the aggregates (1:1), on the other hand, also reached a considerably high compressive strength, that was also due to higher amount of the binder leading to higher density microstructure. However, the obtained mechanical strength of the mortar was still much lower than that of the paste. This can be explained by the properties of the interfacial transition zone (ITZ) between the binder and aggregate particles that in the case of conventional OPC system is considered to be the weakest link that significantly affects the properties of the concrete^36^. However, when comparing the OPC and carbonated CSC mortar, similar to that of this research, Ashraf et al.^7^ states that carbonated CSC mortar system ITZ should be stronger than that of OPC, due to absence of the deposits around the aggregate particles that can dissolve in water causing the formation of a weak zone in the microstructure of the material. Moreover, the ITZ can be affected by the adhesion of the binder to the aggregate particle as well^37^. Paste sample microstructure should be much better bonded, since the system is composed entirely of one material, while in the mortars, the presence of the aggregates that do not react neither with the binder nor the CO_2_, results in weaker bonding and thus weakened microstructure.

According to the obtained compressive strength results it can be seen that it is possible to achieve considerably high results when working with a broad matrix of parameters. Rankinite binder mortar system was able to achieve a compressive strength exceeding 45 MPa. This result was attained by carbonating the mortars at 15 bar CO_2_ pressure for 24 h, at 45 °C, with w/c = 0.25, binder/sand 1:3 and sample compaction with 12.5 MPa.

Even though the determined mechanical properties of the carbonated rankinite binder mortars showed a very favourable and promising results, the long-term durability performance of such system plays a no less important part in pursuing improved alternative binder. Thus, the carbonated samples were exposed to water absorption by immersion, freeze–thaw, and abrasion resistance determination in order to ascertain their durability.

Sample water absorption capability was discussed in a previous paper^38^, the obtained results showed that rankinite binder sample pores were filled with higher amount of carbonation products, than OPC, leading to lower capillary porosity and thus, lower water absorption. Maximum water absorption of rankinite binder samples was equal to 4.14 wt%, OPC sample—5.44 wt%.

After determining the sample capability of water absorption, the same samples were used for the determination of the durability by freeze–thaw resistance using de-icing salt. This method well describes and imitates natural conditions, where concrete is usually exposed to high levels of moisture and temperature changes. Volume expansion in freezing water produces pressure in the pores of the concrete, leading to cavity dilation and rupture that eventually causes expansion and cracking, scaling and crumbling of the concrete. Commonly used de-icing chemicals reduces the freezing point of the precipitation, thus reducing the freezing and thawing cycles^39^. The addition of de-icing salt to water changes the behaviour of the solution during freezing, causing a more gradual formation of the ice^40^. NaCl is the most common de-icing salt used on roadways, due to its comparably low cost^41^. Generally, de-icing salts can alter the degree of saturation or react with the hydrated OPC, thus resulting in expansive reaction products that leads to the development of negative effects on the concrete structure^42^. Solidia Concrete^43^, basically composed of rankinite and wollastonite, has already conducted some durability performance tests and showed that their concrete has significantly better performance in freeze–thaw exposure. However, the mentioned study did not include de-icing salts. Other researchers^44^ have also reported that carbonated calcium silicate based cement can be resistant to the freeze–thaw damage in the presence of CaCl_2_ de-icing salt. Thus, resistance to freeze–thaw cycles using de-icing salts is of high importance, especially when considering the lower temperature climate zones.

**Figure 9:**
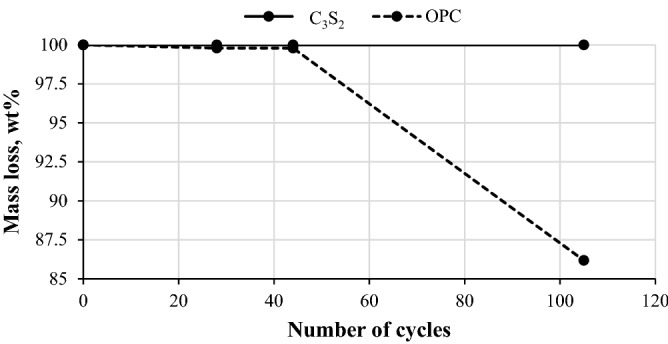
Freeze–thaw mass loss of the carbonated rankinite binder and OPC samples.

**Figure 10:**
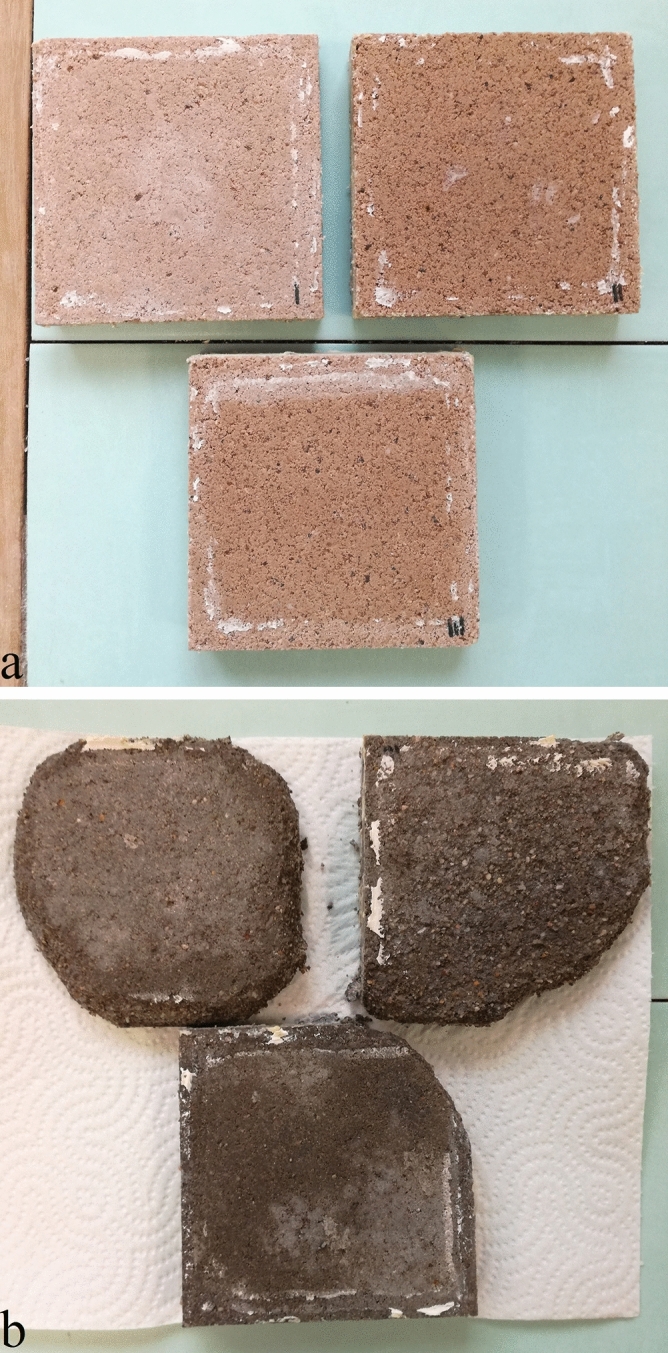
Carbonated rankinite binder (**a**) and OPC (**b**) mortar samples after 105 freeze–thaw cycles.

The obtained results from the durability determination are provided in Fig. 9 where it can be seen that rankinite binder samples showed significantly better performance compared to the OPC samples. Even after more than 100 freeze–thaw cycles the mass of the scaled rankinite binder samples was less than 1 g/m^2^, while for the OPC samples it was significantly higher (5.6 kg/m^2^). Figure 10 portrays the images of rankinite binder and OPC mortars after the 105 freeze–thaw cycles, where it is evident that rankinite binder samples retained their shape, while OPC samples are highly damaged. These results further confirm previous data that lower permeability (lower capillary porosity) hinders concrete saturation during exposure, and the associated reduction in pore sizes restricts ice formation^45^, which leads to lower possibility of scaling and deterioration of the concrete.

Another method to determine concrete durability is abrasion resistance. Wide wheel test method was applied to carbonated rankinite binder mortars and it was determined that after 70 abrasion cycles using abrasive material (corundum) the obtained groove was less than 20 mm wide.

All of the obtained durability results are in compliance with the requirements for concrete paving tiles, according to standard (EN 1338:2003+AC:2006), since water absorption was < 6%, freeze–thaw resistance using de-icing salt was < 1 kg/m^2^, and abrasion resistance was < 20 mm. However, further research considering the long-term performance of the rankinite binder concrete still needs to be conducted and is left to the future investigations.

## Conclusions

In this paper, rankinite binder carbonation hardening process was investigated, regarding the compressive strength development, phase evolution and microstructure, as well as, durability performance. Summarizing the obtained results, the following conclusions were made:

1. During the carbonation hardening process rankinite binder pastes can reach a considerably high compressive strength exceeding 100 MPa during a relatively short period of 24 h making it an attractive alternative binder material in comparison to ordinary Portland cement.
2. The main rankinite binder carbonation reaction products are calcium carbonate, in a form of calcite and aragonite, and silica gel, where calcite is the main component responsible for the binder compressive strength development and final properties of the hardened concrete, while silica gel polymerization does not seem to directly contribute to the strength development.
3. Carbonation process can be described by two stages—phase boundary and diffusion controlled. When the layer of the reaction products reaches a certain degree (or thickness), further reaction is hindered, since CO_2_ can no longer penetrate the dense layer of reaction products and reach the unreacted particle. Thus, even though the sample seems to be carbonated throughout the entire volume, only ~ 40% of the binder particles are reacted.
4. Reaction products forms as a rim around the unreacted particle—first, a layer of silica gel, followed by layer of calcium carbonate that fills the pore structure, thus densifying the microstructure and contributing to compressive strength development.
5. It was determined that rankinite binder is a suitable cementitious material for high strength, highly durable carbonated concrete products that opens a great opportunity for CO_2_ mitigation by permanent sequestration in the concrete structure in the form of stable carbonates and has a high potential for niche applications.
